# Predicting depressive symptoms in employees through life stressors: subgroup analysis by gender, age, working hours, and income level

**DOI:** 10.3389/fpubh.2024.1495663

**Published:** 2024-12-17

**Authors:** Jiwan Moon, Yoosuk An, Sang Won Jeon, Sung Joon Cho

**Affiliations:** ^1^Department of Psychiatry, Kangbuk Samsung Hospital, Sungkyunkwan University School of Medicine, Seoul, Republic of Korea; ^2^Department of Psychiatry, National Traffic Injury Rehabilitation Hospital, Yangpyeong, Republic of Korea; ^3^Department of Neuropsychiatry, Seoul National University Hospital, Seoul, Republic of Korea; ^4^Department of Psychiatry, Seoul National University College of Medicine, Seoul, Republic of Korea; ^5^Workplace Mental Health Institute, Kangbuk Samsung Hospital, Seoul, Republic of Korea

**Keywords:** occupational health, depression, life stress, personnel, regression

## Abstract

**Background:**

Although evidence has accumulated regarding the association between various stressors and depression, few studies have considered the context in which multiple stressors coexist simultaneously. Thus, this study aimed to evaluate the relative importance of seven major life stressors on depressive symptoms: workplace stress, family relationships, interpersonal conflicts, health problems, financial strains, traumatic events, and mannerisms, and analyzed its variation in subgroups.

**Methods:**

Data from 12,541 Korean employees were analyzed. Sociodemographic data such as gender, age, education, marital status, working hours, and income level were collected from the study participants, and the Center for Epidemiologic Studies Depression Scale (CES-D) was employed to assess depressive symptoms. Additionally, life stressors experienced during the previous month and their severity were investigated via a questionnaire. Multiple regression analysis was performed to assess the independent effects of seven major life stressors on depressive symptoms, while controlling for sociodemographic factors. Subgroup analysis was also conducted to determine whether the effect of stressors varied by gender, age, working hours, and income level.

**Results:**

Workplace stress (*β* = 0.411, *p* < 0.001) had the greatest effect on depressive symptoms, followed by mannerisms (*β* = 0.191, *p* < 0.001), family relationships (*β* = 0.120, *p* < 0.001), interpersonal conflicts (*β* = 0.077, *p* < 0.001), health problems (*β* = 0.054, *p* < 0.001), financial strains (*β* = 0.046, *p* < 0.001), and traumatic events (*β* = 0.021, *p* = 0.002). Moreover, significant variance in the rank order of effects of stressors across gender, age, working hours, and income level was observed, as revealed by subgroup analysis.

**Conclusion:**

This study identified the effects of seven major life stressors on depressive symptoms and suggests that the rank order of these effects varies depending on sociodemographic factors. These findings expand the understanding of the complex relationship between concurrent life stressors and depression, and highlight the need for personalized interventions to prevent and manage depression among Korean employees.

## 1 Introduction

There is accumulating evidence that environmental factors are significant predictors of the development of depression (1), and that stressful events can trigger depression (2–5). Particularly, studies have reported that the first depressive episode is intimately associated with major life stress (6–8). Depression adversely impacts work outcomes (9), resulting in significant societal costs (10). Therefore, preventing and managing depression in the workforce is crucial not only for individuals, but also for public health.

It is well recognized that workplace stress is a major predictor of depression (5, 11–13); however, research on the effects of stressors other than workplace stress on depression remains scarce (12, 14–16). For example, a study using health screening data analyzed the association between the top stressors during the previous month and a diagnosis of depression (16). However, studies have often ignored the fact that employees may experience multiple stressors concurrently, and that the severity of stress may differ in terms of the magnitude of effect on depressive symptoms. Furthermore, researchers have not sufficiently considered other factors that may influence depressive symptoms, such as education, marital status, working hours, and income level. To accurately ascertain the relationship between stress and depressive symptoms, it is necessary to assess the severity of each stressor and evaluate even subclinical depressive symptoms using depression scale scores, rather than distinguishing the presence or absence of depression. These analyses should be conducted while controlling for factors that may affect depressive symptoms.

Meanwhile, recent studies suggest that the effects of stress on depressive symptoms may vary according to various sociodemographic factors. For example, it has been reported that the association between workplace stress and depression is stronger in females (11). Regarding age differences, the association between negative stress and depression is lower among the older adult compared to younger adults, and a few studies have shown that depression in the older adult is better explained by physical illness burden (17, 18). Additionally, the most severe stressors vary by gender and age (16). While there is limited research on the effect of working hours and income level on the relationship between stress and depression, it is evident that each of these factors has a significant effect on depression. Extended work hours have been associated with depression, particularly among females and low-income employees (19). The relationship between income level and depression has been reported to vary across studies (20, 21), but financial stress itself—independent of objective income level—has been identified as a strong predictor of depression (22). However, very few studies have systematically analyzed the effect of sociodemographic factors on the relationship between various stressors and depression.

This study aims to compare the effects of seven major life stressors on depressive symptoms. Furthermore, it aims to determine how the rank order of effect of these life stressors on depressive symptoms varies by gender, age group, working hours, and income level. The ultimate goal of this research is to contribute to the development of more sophisticated depression prevention and intervention strategies.

## 2 Methods

### 2.1 Study participants

Data were collected from employees aged 19–65 years who received workplace mental health screenings at the Workplace Mental Health Institute of Kangbuk Samsung Hospital (Seoul, Republic of Korea) between April 2020 and November 2022. A total of 12,565 subjects completed the questionnaire, and after applying purposive sampling to exclude incomplete responses, a total of 12,541 subjects were selected for the study. The selected study participants included employees from 23 private or public organizations.

As the study utilized anonymized data collected via routine health screenings, requirement for consent from the selected participants was waived. All study procedures were approved by the Institutional Review Board of Kangbuk Samsung Hospital (KBSMC 2022–03-046) and conducted in accordance with the Declaration of Helsinki.

### 2.2 Assessments

The data regarding gender, age, education level, marital status, and average working hours per week over the previous year were collected. Additionally, participants selected an income bracket corresponding to their monthly salary over the previous year, which was then categorized into three groups: low income (<200 million KRW), middle income (200–400 million KRW), and high income (≥400 million KRW).

Depressive symptoms were assessed using the Korean version of the Center for Epidemiological Studies Depression Scale (CES-D). The CES-D is a self-report scale, comprising 20 questions on a Likert scale ranging from 0 to 3 points. The total score is 60 points, with higher scores indicating more severe depressive symptoms (23, 24). The Korean version of the CES-D has been confirmed to have desirable internal consistency (Cronbach’s *α* = 0.977) (25).

To assess the stressors for the study participants, the following seven factors were selected as major life stressors based on the stress questionnaire within the Korea National Health and Nutrition Examination Survey (26): (1) workplace stress (excessive workload, conflicts with bosses or coworkers, missing promotions, etc.), (2) family relationships (conflicts with family members, changes in the family such as deaths and births, marriages, and divorces), (3) interpersonal conflicts (conflicts with people around study participants such as friends and lovers), (4) health problems (illnesses, injuries, etc.), (5) financial strains, (6) traumatic events (traffic accidents, physical and sexual assaults, crimes, accidental events, etc.), and (7) mannerisms (feeling lethargic and bored because of lack of change in daily life). Participants were asked to select all life stressors that had caused them stress during the previous month, and to rate the extent to which they interfered with their daily lives on a natural number scale from 0 to 100. The scale ranged from 0 for not interfering at all to 100 for interfering so severely that they were unable to do anything about it.

### 2.3 Statistical analyses

Sociodemographic and clinical characteristics were presented as means and standard deviations or frequencies using descriptive analyses. Differences in the degree of effects of the seven major life stressors on depressive symptoms were analyzed using multiple regression analysis. This analysis was conducted in two steps: in the first step, sociodemographic factors such as gender, age, education, marital status, income level, and working hours were included as independent variables and treated as covariates, and in the second step, scores on each of the seven factors were included as independent variables. The standardized coefficient beta values were mutually compared, based on which the different effects of stressors on depressive symptoms assessed by CES-D scores were compared. Furthermore, subgroup analysis was conducted to compare the differences in the effect of stressors on depressive symptom scores by gender, age group, working hours, and income level. Four age groups were determined: under 30, 30s, 40s, and 50+. Considering that the Labor Standards Act of South Korea defines the statutory working hours per week as 40 h, and up to 52 h can be worked if 12 h of overtime are included, three groups were set for working hours: 40 h or less, over 40 h but not more than 52 h, and over 52 h. Three groups of income level, categorized while collecting data as a sociodemographic factor, were retained in order to explore whether there were differences in the effects of stressors on depression scores across groups. All statistical analyses were performed using the Statistical Package for the Social Sciences (SPSS) v23.0 for Windows.

## 3 Results

### 3.1 Sociodemographic and clinical characteristics of study participants

The sociodemographic and clinical characteristics of the study participants are presented in Table 1. There were more men in the study (62.9% vs. 37.1%) than females, with an average age of 36.7 years (SD 9.1). The mean CES-D score was 15.0 (SD 10.1), and among the seven life stressors, workplace stress had the highest mean score of 32.6 (SD 29.8) and traumatic events had the lowest mean score of 3.2 (SD 11.8).

**Table 1:** Sociodemographic and clinical characteristics of the study participants (*N* = 12,541).

Characteristic	
Sex, *n* (%)	
Male	7,889 (62.9)
Female	4,652 (37.1)
Age (years), mean ± SD	36.7 ± 9.1
Education (graduate), *n* (%)	
College graduate or below	2,911(23.2)
Bachelor’s degree	7,742 (61.7)
Master’s degree or higher	1,888(15.1)
Marital status, *n* (%)	
Single	5,438 (43.3)
Married	6,857 (54.7)
Other^a^	246(2.0)
Working hour (weekly, hour), mean ± SD	47.0 ± 7.6
Income (monthly), *n* (%)	
Low income	537 (4.3)
Middle income	6,710 (53.5)
High income	5,294 (42.2)
CES-D score, mean ± SD	15.0 ± 10.1
Workplace stress, mean ± SD	32.6 ± 29.8
Family relationships, mean ± SD	13.7 ± 22.0
Interpersonal conflicts, mean ± SD	9.3 ± 17.8
Health problems, mean ± SD	15.0 ± 21.6
Financial strains, mean ± SD	15.8 ± 22.8
Traumatic events, mean ± SD	3.2 ± 11.8
Mannerism, mean ± SD	20.0 ± 25.0

SD, standard deviation; CES-D, Center for Epidemiological Studies Depression Scale.

a Include separated, divorced, widowed.

### 3.2 Multiple regression analysis

The results of the multiple regression analysis on the effects of sociodemographic factors and the seven life stressors on depressive symptoms are presented in Table 2. Both Models 1 and 2 were found to be significant.

**Table 2:** Multiple regression analysis of factors predicting depressive symptoms (*N* = 12,541).

Predictor	Model 1				Model 2				
	*B*	*β*	*t*	*p*	*B*	*β*	*t*	*p*	*VIF*
Sex (reference: male)	3.695	0.176	19.743^***^	<0.001	1.330	0.063	9.066^***^	<0.001	1.123
Age	0.038	0.034	2.963^**^	0.003	0.012	0.010	1.156	0.248	1.876
Education (reference: bachelor’s degree)									
College graduate or below	−0.932	−0.039	−4.325^***^	<0.001	0.302	0.013	1.819	0.069	1.098
Master’s degree or higher	−1.060	−0.037	−4.111^***^	<0.001	−0.476	−0.017	−2.409^*^	0.016	1.120
Marital status (reference: single)									
Married	−1.570	−0.077	−6.818^***^	<0.001	−1.215	−0.060	−6.763^***^	<0.001	1.789
Other^a^	2.344	0.032	3.534^***^	<0.001	1.121	0.015	2.190^*^	0.029	1.127
Working hour (weekly)	0.166	0.124	14.315^***^	<0.001	0.036	0.027	3.999^***^	<0.001	1.055
Income (monthly, reference: middle income)									
Low income	2.337	0.047	5.267^***^	<0.001	1.876	0.038	5.512^***^	<0.001	1.062
High income	−0.848	−0.041	−4.098^***^	<0.001	−0.541	−0.026	−3.400^**^	0.001	1.380
Workplace stress					0.139	0.411	53.477^***^	<0.001	1.351
Family relationships					0.055	0.120	15.744^***^	<0.001	1.335
Interpersonal conflicts					0.044	0.077	10.238^***^	<0.001	1.300
Health problems					0.025	0.054	7.016^***^	<0.001	1.342
Financial strains					0.020	0.046	5.958^***^	<0.001	1.375
Traumatic events					0.018	0.021	3.041^**^	0.002	1.080
Mannerism					0.077	0.191	25.263^***^	<0.001	1.305
*R* ^2^	0.069				0.453				
Adjusted *R*^2^	0.068				0.452				
*R*^2^ change	0.069				0.384				
*F*	102.433^***^				647.928^***^				
*F* change	102.433^***^				1256.884^***^				

VIF, variance inflation factor.

a Include separated, divorced, widowed.

*p < 0.05, **p < 0.01, ***p < 0.001.

In Model 1, females were more depressed than men (*β* = 0.176, *p* < 0.001). Additionally, depression exacerbated with age (*β* = 0.034, *p* = 0.003) and extended working hours (*β* = 0.124, *p* < 0.001).

In Model 2, among the seven major life stressors, workplace stress had the strongest effect on CES-D scores (*β* = 0.411, *p* < 0.001), followed by mannerisms (*β* = 0.191, *p* < 0.001), family relationships (*β* = 0.120, *p* < 0.001), interpersonal conflicts (*β* = 0.077, *p* < 0.001), health problems (*β* = 0.054, *p* < 0.001), and financial strains (*β* = 0.046, *p* < 0.001) in order. Traumatic events had the lowest effect (*β* = 0.021, *p* = 0.002).

### 3.3 Subgroup analysis

The results of the subgroup analysis are presented in Table 3. Both Models 1 and 2 were found to be significant in all subgroups.

**Table 3:** Subgroup analysis of stressors predicting depressive symptoms (*N* = 12,541).

Subgroup	Prediction order (*β*)						
	1 (most predictive)	2	3	4	5	6	7
Sex							
Male	Workplace stress (0.409^***^)	Mannerism (0.208^***^)	Family relationships (0.109^***^)	Interpersonal conflicts (0.079^***^)	Financial strains (0.071^***^)	Health problems (0.044^***^)	Traumatic events (0.007)
Female	Workplace stress (0.411^***^)	Mannerism (0.169^***^)	Family relationships (0.142^***^)	Interpersonal conflicts (0.080^***^)	Health problems (0.075^***^)	Traumatic events (0.041^***^)	Financial strains (0.006)
Age							
<30	Workplace stress (0.407^***^)	Mannerism (0.188^***^)	Interpersonal conflicts (0.132^***^)	Family relationships (0.094^***^)	Health problems (0.074^***^)	Traumatic events (0.035^**^)	Financial strains (0.032^*^)
30s	Workplace stress (0.415^***^)	Mannerism (0.203^***^)	Family relationships (0.124^***^)	Interpersonal conflicts (0.078^***^)	Financial strains (0.047^***^)	Traumatic events (0.044^***^)	Health problems (0.027^*^)
40s	Workplace stress (0.395^***^)	Mannerism (0.163^***^)	Family relationships (0.130^***^)	Health problems (0.060^***^)	Financial strains (0.058^***^)	Interpersonal conflicts (0.044^**^)	Traumatic events (0.001)
50+	Workplace stress (0.373^***^)	Mannerism (0.196^***^)	Family relationships (0.139^***^)	Health problems (0.092^***^)	Financial strains (0.048)	Traumatic events (−0.005)	Interpersonal conflicts (−0.006)
Working hour							
≤40	Workplace stress (0.345^***^)	Mannerism (0.192^***^)	Family relationships (0.186^***^)	Interpersonal conflicts (0.081^***^)	Health problems (0.061^**^)	Financial strains (0.020)	Traumatic events (0.009)
41 ~ 52	Workplace stress (0.409^***^)	Mannerism (0.195^***^)	Family relationships (0.113^***^)	Interpersonal conflicts (0.080^***^)	Financial strains (0.055^***^)	Health problems (0.050^***^)	Traumatic events (0.011)
>52	Workplace stress (0.449^***^)	Mannerism (0.184^***^)	Family relationships (0.084^***^)	Traumatic events (0.074^***^)	Interpersonal conflicts (0.064^**^)	Health problems (0.055^**^)	Financial strains (0.040)
Income							
Low	Workplace stress (0.452^***^)	Mannerism (0.136^***^)	Family relationships (0.120^**^)	Health problems (0.105^**^)	Interpersonal conflicts (0.087^*^)	Traumatic events (0.073^*^)	Financial strains (0.028)
Middle	Workplace stress (0.416^***^)	Mannerism (0.194^***^)	Family relationships (0.116^***^)	Interpersonal conflicts (0.102^***^)	Health problems (0.051^***^)	Financial strains (0.042^***^)	Traumatic events (0.019^*^)
High	Workplace stress (0.399^***^)	Mannerism (0.193^***^)	Family relationships (0.130^***^)	Health problems (0.055^***^)	Financial strains (0.055^***^)	Interpersonal conflicts (0.039^**^)	Traumatic events (0.019)

*p < 0.05, **p < 0.01, ***p < 0.001.

#### 3.3.1 Difference by gender

In the multiple regression analysis of female employees (*N* = 4,652), the effect of financial strains on depressive symptoms was not statistically significant (*p* = 0.626), and the rank order of effects of other stressors was the same as that of all employees. Conversely, among male employees (*N* = 7,889), financial strains (*β* = 0.071, *p* < 0.001) were a better predictor of depressive symptoms than health problems (*β* = 0.044, *p* < 0.001). The effect of traumatic events on depressive symptoms was not significant (*p* = 0.401).

#### 3.3.2 Difference by age group

For those under 30 (*N* = 3,339), interpersonal conflicts (*β* = 0.132, *p* < 0.001) were better predictors of depressive symptoms than family relationships (*β* = 0.094, *p* < 0.001), and traumatic events (*β* = 0.035, *p* = 0.006) were also better predictors of depression than financial strains (*β* = 0.032, *p* = 0.023). In the group of 30s (*N* = 4,678), financial strains (*β* = 0.047, *p* < 0.001) and traumatic events (*β* = 0.044, *p* < 0.001) were found to be better predictors than health problems (*β* = 0.027, *p* = 0.028). In the group of 40s (*N* = 3,166), health problems (*β* = 0.060, *p* < 0.001) and financial strains (*β* = 0.058, *p* < 0.001) had greater predictive power than interpersonal conflicts (*β* = 0.044, *p* = 0.006), while traumatic events were not significant (*p* = 0.931). Among those aged 50+ (*N* = 1,358), financial strains (*p* = 0.059), traumatic events (*p* = 0.818), and interpersonal conflicts (*p* = 0.798) did not have a significant effect on depressive symptoms, and the rank order of effects of other stressors was the same as among all employees.

#### 3.3.3 Difference by working hours

In the group working 40 h or less per week (*N* = 2,481), predictors of depressive symptoms were in the same order for all employees and life stressors, while financial strains (*p* = 0.271) and traumatic events (*p* = 0.564) were not significant predictors. In the group working more than 40 h but less than 52 h per week (*N* = 8,359), financial strains (*β* = 0.055, *p* < 0.001) were a better predictor of depressive symptoms than health problems (*β* = 0.050, *p* < 0.001), and traumatic events had no significant effect (*p* = 0.186). In the group working more than 52 h per week (*N* = 1701), the three lowest stressors predicting depressive symptoms were traumatic events (*β* = 0.074, *p* < 0.001), interpersonal conflicts (*β* = 0.064, *p* = 0.002), and health problems (*β* = 0.055, *p* = 0.007), while financial strains were not a significant predictor (*p* = 0.062).

#### 3.3.4 Differences by income level

Among low-income employees (*N* = 537), health problems (*β* = 0.105, *p* = 0.004) were a better predictor of depressive symptoms than interpersonal conflicts (*β* = 0.087, *p* = 0.021), while financial strains (*p* = 0.447) had no significant effect. Among middle-income employees (*N* = 6,710), the rank order of effects of stressors was the same as among all employees. Among high-income employees (*N* = 5,294), the top-three life stressors that had the greatest effects on the CES-D score were the same as among all employees, but then, health problems (*β* = 0.055, *p* < 0.001), financial strains (*β* = 0.055, *p* < 0.001), and interpersonal conflicts (*β* = 0.039, *p* = 0.001) had the greatest predictive power for depressive symptoms. Traumatic events had no significant effect (*p* = 0.088).

## 4 Discussion

This study systematically analyzed the effects of seven major life stressors on depression in employees, and examined differences in the rank order of effects of stressors according to various sociodemographic factors such as gender, age group, working hours, and income level. The strength of this study is that the analyses included the effects of each stressor on subclinical depressive symptoms using CES-D scores. In previous studies, the presence or absence of specific stressors or the severity scores of each stressor were compared between depressed patient groups and control groups (12, 14). In other studies, simplistic analyses were conducted to ascertain whether the presence or absence of a stressor contributes to the presence or absence of depression (15, 16). However, these approaches were insufficient for comparing the relative effects of life stressors, and ignored the contribution of individual stressors to subclinical depressive symptoms.

This study identified that all life stressors affect depressive symptoms in the entire study population. Workplace stress, mannerisms, family relationships, interpersonal conflicts, health problems, financial strains, and traumatic events have the greatest effect on depressive symptoms, in that order. Many studies have shown that workplace stress predicts depression (5, 11–13, 15, 16), and that not only acute work-related stress but also structural occupational factors associate with depression (27). Employees of all age groups and genders also chose workplace stress as the most stressful factor (16). Therefore, considering that the study participants were employees, it is natural that workplace stress is the best predictor of depressive symptoms. Additionally, studies have reported an association between mannerisms and depression (15, 16), which could be caused by repetitive tasks and routines in employees. Family relationships (12, 15, 16), interpersonal conflicts (12, 16, 28), health problems (12, 14, 16), financial strains (12, 14, 16, 22, 29, 30), and traumatic events (31) are also known to be associated with the onset of depression. Conversely, a few studies have reported different rank orders of effects of stressors compared to this study, such as interpersonal stress better predicting depression than non-interpersonal stress (28), health problems having the greatest effect on the onset of depression (12), or stress due to changes in relationships having the highest effect in increasing the probability of the development of depressive symptoms (16). However, as these studies utilized fewer categories of stressors than this study (12, 28), or had differences in the classification of stressors (16), simple comparisons are inadvisable. Additionally, there were limitations in the study design, such as comparing the effects of stressors merely based on the difference in scale scores between the depression and control groups (12) or calculating the odds of developing depression for a specific stressor without considering other stressors (16). The rank order of effects of various life stressors on depressive symptoms, derived from this study, reflects the context in which stressors collectively affect depressive symptoms, making it a unique and meaningful finding.

Furthermore, the study explored how the rank order of effects of life stressors varies according to sociodemographic factors. In the case of male employees, financial strains had a greater effect when compared to the overall study participants. This may be related to the cultural background of South Korea, which still emphasizes the economic support role of males (32). Economic stability may have a greater effect on depressive symptoms in male employees than in female employees because it is linked to social recognition and self-esteem. In fact, research has shown that unemployment tended to lower self-esteem in males and lead to depressive symptoms, but this association was not identified in females (33).

Among employees under 30 years of age, interpersonal conflicts had a greater impact on the occurrence of depressive symptoms than family relationships. According to Erikson’s theory regarding the stages of psychosocial development, young adults experience the stage of intimacy versus isolation (34). Additionally, this is the period of emerging adulthood, when young adults leave their families and form a personal identity (35). The significance of interpersonal conflicts increases more than in other age groups because social support is associated with identity exploration (36). In fact, stress in interpersonal relationships has been reported as the most stressful factor after work stress among both males and females in their 20s (16). Meanwhile, given that trauma can influence an individual’s identity (37), the fact that traumatic events are stronger predictors of depression than financial strains among employees under 30 may be also related to this period being crucial for identity formation.

Among employees in the age groups of 30s and 40s, the significance of financial issues increased. This is consistent with previous research findings reporting that financial strains were the third most stressful factor for males in their 30s and 40s (16). As individuals in this age group have to be financially independent, form a family, and maintain life stability (30), they are more likely to experience stress than other age groups if they experience financial issues, which is highly likely to be linked to depressive symptoms. Meanwhile, traumatic events ranked higher in the age group of 30s than in entire study population. A previous study reported that unusual happenings are not a frequently reported stress factor in the age group of 30s (16). Thus, it would be natural to interpret that those in their 30s are more affected by such events rather than because they occur more frequently in that age group. The 30s is a period of established adulthood, during which individuals should meet simultaneous demands such as career development, maintaining intimate relationships, and raising children (38). Given that traumatic events affect an individual’s sense of control (39), the loss of control in their 30s, when these complex roles are required, can cause greater stress, inducing depressive symptoms. The effect of health problems on depressive symptoms increased in the age group of 40s, which is consistent with previous findings that chronic diseases are significant determinants of depression among the middle-aged and the older adult (40). Moreover, as midlife is a period of generativity versus stagnation (34), health problems during this period may not only be stressful in themselves but also in terms of hindering an individual’s generativity.

In the group of employees whose weekly working hours exceeded the statutory working hours of 40 h but were less than the maximum extended working hours of 52 h, financial strains were better predictors of depressive symptoms than health problems. It is possible that employees in this group experienced greater financial stress because of the imbalance between overtime work and the financial compensation expected. According to the effort–reward imbalance theory, psychological stress increases and depression can occur when employees do not receive adequate reward for their efforts (41). In fact, a study reported that employees who did not receive appropriate reward for overtime work had higher burnout scores (42).

In the group with working hours exceeding 52 h per week, traumatic events had a greater effect on depressive symptoms than interpersonal conflicts and health problems. Extended working hours are associated with low resilience (43), and resilience mitigates the effect of traumatic events on depression (44). Therefore, employees with extended working hours may be more vulnerable to traumatic events owing to low resilience. Studies showing that extended working hours exacerbate employees’ mental health support this hypothesis (19, 45). While social support is a key factor in mitigating the effects of traumatic events on depression (44), extended working hours reduce the time available for family and friends, leading to reduced opportunities for social support. This is another reason why employees with extended working hours may be more vulnerable to traumatic events.

Regarding differences by income level, health problems better predicted depressive symptoms among low-income employees than interpersonal conflicts compared to that among all employees. This is because low-income employees are less likely to use preventive medical services because of financial strains (46), and generally, they have a higher likelihood of exposure to unstable work environments, which can induce frequent health problems (47). Additionally, health problems may cause greater stress to low-income employees because access to medical services is limited owing to economic difficulties (46, 48).

Among high-income employees, health problems and financial strains have a greater impact on depressive symptoms than interpersonal conflicts. This may reflect the fact that high-income employees are more concerned about economic stability and have higher levels of interest in health because of greater healthcare accessibility. Additionally, they often have higher responsibilities and higher workloads, which can lead to more frequent health problems. Thus, the impact of health problems may remain great (49).

This study contributes to expanding the understanding of the complex etiology of depression by examining whether the rank order of effects of stressors varies according to various sociodemographic factors. The findings provide new perspectives on depression prevention strategies. In particular, the research indicates that rather than applying the identical stress management techniques for all employees, a more effective strategy would be to implement tailored methods that focus on life stressors relevant to individual characteristics. For instance, since interpersonal conflicts significantly affect depressive symptoms in employees under 30, workshops aimed at enhancing interpersonal skills and organizing workplace networking activities may be beneficial in fostering social support and, in turn, preventing depression. On the other hand, for employees in their 30s and 40s, where financial strains are a more significant predictor of depressive symptoms, interventions such as workplace financial counseling, financial management seminars, and access to low-interest loan programs could be more effective in preventing depression. These tailored strategies are practical and have the potential to alleviate stress among employees. However, given the multifaceted nature of individuals, it is essential to recognize that personalized interventions should account for a comprehensive integration of various individual characteristics rather than relying solely on a single sociodemographic factor. Such interventions could contribute not only to the prevention and management of depression but also to improving productivity among employees (9) and reducing the social costs of depression (10).

The limitations of this study are as follows. First, this was designed as a cross-sectional study, which made it difficult to conclude the causal relationships between the associations identified in multiple regression analyses. However, considering a previous study showing that stress events precede depressive episodes (6), it could be speculated that stress factors are likely to be associated with the occurrence of depressive symptoms. Second, the nature of self-report questionnaires may affect the validity of the results and requires careful interpretation. In our study, study participants were asked to recall life stressors that had occurred within the previous month at the time they completed the questionnaires, which could introduce recall bias. For example, life stressors that occurred more recently might be reported with greater severity, potentially leading to either underestimation or overestimation of the contribution of each life stressor to depressive symptoms. In addition, because our study used data from health screenings that were required by the study participants’ affiliated institutions, and our questionnaires addressed sensitive and personal topics, social desirability bias could be introduced. This may have led to an underestimation of the impact of some life stressors. Therefore, further studies should include the objective measures of life stressors to adjust for the subjective assessments of study participants and reduce potential bias. For example, financial strain could be assessed with objective financial status. It may also be helpful to use a peer or family member’s assessment of a study participant’s stress score. Third, although the questionnaire employed in this study was constructed based on previously validated scales (26, 50), the validity of the questionnaire itself was not established in this study. Fourth, the data collected in this study were limited to the severity of subjective discomfort caused by stress, and a nuanced investigation of specific stressors (e.g., specific stressors about family members) was not conducted. Future research would require in-depth investigations of these specific factors. Fifth, as this study only investigated stressors within the previous month in self-report questionnaires, the impact of acute and chronic stress on depressive symptoms may differ (7). Therefore, it will be crucial to distinguish and investigate these factors in subsequent studies. Sixth, risk factors for the development of depressive symptoms, such as a family history of depression, were not sufficiently considered in this study. Therefore, caution is required when interpreting the results. Finally, while the relatively large sample size compared to previous studies may strengthen the generalizability of the findings, the exclusive focus on Korean employees remains a limitation that may limit the applicability of the results. Given that culture has a significant impact on stress and coping paradigms (51), it is necessary to verify that similar results are obtained among employees from different countries and cultures in future research.

In conclusion, this study identified the rank order of effects of seven major life stressors on depression in Korean employees. Additionally, it examined how the rank order of effect of these stressors varies depending on various sociodemographic factors. The results suggest the need for personalized interventions tailored to individual characteristics for the prevention and management of depression among employees.

## Data Availability

The original contributions presented in the study are included in the article, further inquiries can be directed to the corresponding authors.
